# Unraveling the Role of RNase L Knockout in Alleviating Immune Response Activation in Mice Bone Marrow after Irradiation

**DOI:** 10.3390/ijms25052722

**Published:** 2024-02-27

**Authors:** Kexin Ding, Hujie Li, Fumin Tai, Junzhao Duan, Qiong Wang, Rui Zhai, Hanjiang Fu, Changhui Ge, Xiaofei Zheng

**Affiliations:** Beijing Key Laboratory for Radiobiology, Department of Experimental Hematology and Biochemistry, Beijing Institute of Radiation Medicine, Beijing 100850, China; cushingding@163.com (K.D.); lihujie0309@163.com (H.L.); taifm810@163.com (F.T.); duanjz97@163.com (J.D.); doitnow2021@163.com (Q.W.); zhairui0128@163.com (R.Z.); fuhj75@126.com (H.F.)

**Keywords:** RNase L, irradiation, bone marrow, immune response

## Abstract

Ionizing radiation (IR) induces severe hematopoietic injury by causing DNA and RNA damage as well as activating the immune responses, necessitating the development of effective therapeutic strategies. Ribonuclease L (RNase L) as an innate immune response pathway is triggered by exogenous and endogenous abnormal dsRNA under viral infection and dyshomeostasis, thereby activating the immune responses. Thus, we investigated the effect of RNase L on irradiation-induced bone marrow damage using RNase L knockout (RNase L^−/−^) mice. Phenotypic analysis revealed that RNase L knockout mitigates irradiation-induced injury in the bone marrow. Further investigation into the mechanism of RNase L by RNA-seq, qRT-PCR, and CBA analysis demonstrated that RNase L deficiency counteracts the upregulation of genes related to immune responses induced by irradiation, including cytokines and interferon-stimulated genes. Moreover, RNase L deficiency inhibits the increased levels of immunoglobulins in serum induced by irradiation. These findings indicate that RNase L plays a role in the immune response induced by irradiation in the bone marrow. This study further enhances our understanding of the biological functions of RNase L in the immune response induced by irradiation and offers a novel approach for managing irradiation-induced bone marrow injury through the regulation of RNase L activation.

## 1. Introduction

The advancement of science and technology has significantly improved the utilization of IR and non-ionizing radiation (NIR) in various fields, including medicine and industry. However, the potential hazards associated with exposure to both IR and NIR remain. Extensive research has demonstrated that exposure to IR and NIR can have diverse effects on multiple biological systems, such as the hematopoietic [1,2] and central nervous systems [3,4]. NIR exerts a profound influence on the central nervous system by inducing the production of reactive oxygen species (ROS). Different doses of total body irradiation (TBI) cause acute radiation syndrome (ARS), which can be categorized into three sub-syndromes: hematopoietic ARS (H-ARS), gastrointestinal ARS, and neurovascular ARS. IR causes severe damage to cells and tissues through direct mechanisms such as the induction of DNA double-strand breaks (DSBs), an increase in ROS, and indirect mechanisms such as the upregulation of inflammatory factors leading to a cytokine storm and subsequent hyperactivated immune responses [5,6]. Interestingly, existing studies have reported that the SARS-CoV-2 infection triggers inflammation and immune responses similar to those triggered by irradiation [7,8]. Both SARS-CoV-2 infection and irradiation exposure can cause damage to multiple organs in the body, including the immune system, hematopoietic system, lung, liver, and so on. This can be attributed to the induction of a systemic cytokine storm, characterized by the activation of proinflammatory cytokines such as interferon alpha (IFNA), interferon beta (IFNB), interleukin 1 beta (IL1B), interleukin 6, interleukin 18 (IL18), as well as the disruption of lymphocyte population and function [7]. Consequently, hyperactivated inflammation and dysregulated immune responses emerge as shared underlying factors in the development of multi-tissue injuries caused by SARS-CoV-2 infection and irradiation exposure. The intersection between viral infection and ARS could accelerate the development of new treatment strategies for each other.

Several intracellular DNA and RNA sensors, essential for antiviral infection, have been identified as key players in irradiation-induced injury, presenting potential targets for mitigating and managing acute radiation injury. The cyclic GMP-AMP synthase (cGAS) and stimulator of interferon response cGAMP interactor (STING) pathway is activated by both endogenous and exogenous double-stranded DNA (dsDNA) and subsequently regulates the immune responses [8]. The activation of the cGAS-STING pathway has been shown to suppress SARS-CoV-2 replication via recognizing cytoplasmic chromatin of infected cells [9] and also enhances the effects of irradiation by controlling ROS homeostasis and DNA damage [10]. Additionally, multiple RNA sensing pathways have been reported to have synergistic effects in resisting viral infection by recognizing both endogenous and exogenous double-stranded RNA (dsRNA), including RNA sensor RIG-1 (RIG1) and mitochondrial antiviral signaling protein (MAVS) pathway [11], 2′-5′-oligoadenylate synthetase (OAS) and RNase L pathway [12], and others. These pathways recognize endogenous RNA induced by genotoxic stress as if they are the exogenous RNA pathogen [13]. Specifically, the RIG1-MAVS pathway is activated when mitochondrial RNA is released into the cytoplasm leading to the initiation of an immune response in the presence of irradiation-induced mtDNA damage [14]. However, the impact of RNase L on irradiation-induced damage remains uncertain.

The OAS-RNase L pathway, an essential innate immune response pathway against pathogenic microorganisms, particularly viruses, is triggered by dsRNA and IFN signals [15,16]. RNase L, which is activated by 2′-5′-linked oligoadenylate produced by OAS, inhibits viral infections by cleaving the single-stranded RNA of viruses and cells [17]. These degradation products of RNA can activate pathogen-associated molecular patterns and endogenous damage-associated molecular patterns recognition receptors, then mediate the expression of downstream inflammatory factors (e.g., IFNB, IL1B, IL18) to resist viral infection [18,19,20,21]. Subsequently, IFNB influences the processes of immunoglobulin class switching in B cells, and the activation of T cells and NK cells, thereby modulating the adaptive immune response against viral infection [22,23]. Mechanistically, RNase L reprograms transcription and translation by inducing extensive degradation of intracellular RNA, while also facilitating the transcription and translation of antiviral genes like IFN during viral infections [24,25]. Furthermore, several studies have reported the activation of RNase L by endogenous abnormal dsRNA [26,27,28,29]. For instance, the overexpression of specificity protein 1 results in the production of small self-RNAs that activate the OAS-RNase L pathway [26]. The deprivation of oxygen and glucose induces the abnormal aggregation of dsRNA, activating RNase L, and facilitating apoptosis in H9c2 cells [27]. The alteration of RNA N6-methyladenosine modifications induces the formation of endogenous dsRNAs, subsequently activating RNase L and causing an aberrant innate immune response and hematopoietic failure [28]. Folate deficiency leads to the accumulation of endogenous dsRNA, thereby upregulating and activating the OAS pathway [29]. In summary, the OAS-RNase L pathway plays a critical role in pathogenic microorganism invasion and dyshomeostasis without pathogens.

Therefore, it is hypothesized that the activation of the RNase L pathway, resulting from RNA damage caused by irradiation, plays a significant role in the irradiation response. The RNase L pathway is an important innate immune response pathway, and the hematopoietic and immune systems are highly sensitive to irradiation. H-ARS, resulting from severe injury to the hematopoietic system, emerges as a primary cause of mortality after moderate and high doses of TBI [1]. As a crucial component of the hematopoietic and immune systems, the bone marrow is particularly susceptible to irradiation-induced injury [30]. IR directly causes harm to hematopoietic stem cells and lymphocyte subpopulations within the bone marrow [31]. Consequently, the development of effective treatment strategies for acute hematopoietic radiation injury remains a pressing concern. This study aims to investigate the impact of the RNase L pathway on irradiation-induced damage in the bone marrow using the RNase L^−/−^ mice. We found RNase L deficiency partially mitigates irradiation-induced bone marrow injury in mice. Mechanistically, RNase L deficiency alleviates the irradiation-induced immune response by modulating the expression of cytokines, interferon-stimulated genes, and immunoglobulins. These findings highlight the involvement of RNase L in the immune response induced by irradiation in the bone marrow. This study expands our understanding of the biological roles of RNase L in irradiation-induced bone marrow damage, thereby offering a novel therapeutic target for H-ARS by modulating the activation of RNase L.

## 2. Results

### 2.1. Irradiation-Induced Body Injury Is Partially Relieved in RNase L^−/−^ Mice

**Figure 1:**
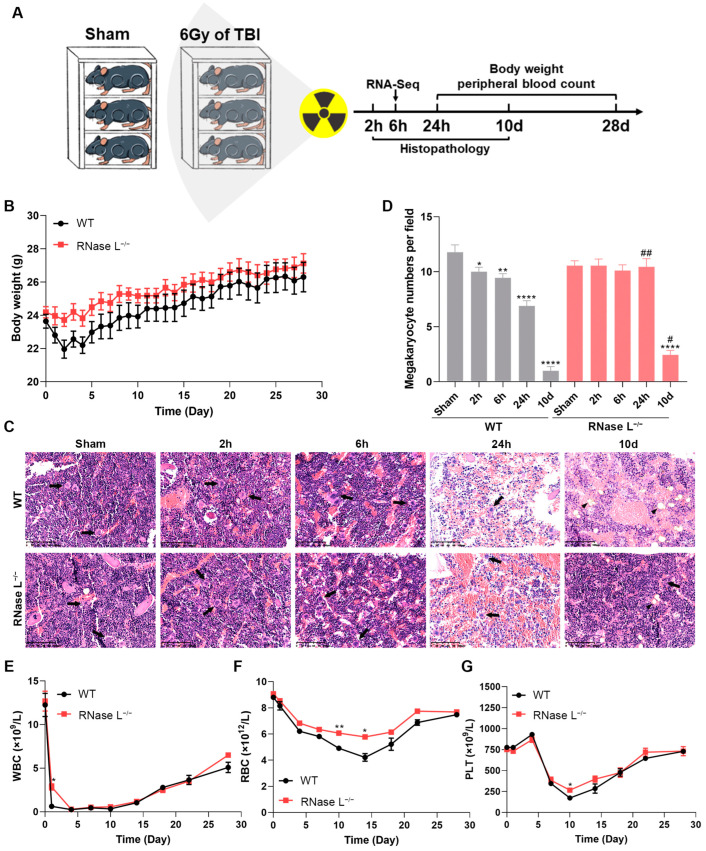
RNase L knockout partially relieves the irradiation-induced body injury in mice. (**A**) Schematic diagram of the irradiation exposure. (**B**) Body weight of the WT and RNase L^−/−^ mice treated with 6 Gy of TBI (*n* = 5). (**C**) Representative images of hematoxylin and eosin (H&E)-stained femurs of the WT and RNase L^−/−^ mice treated with 6 Gy of TBI or sham-irradiation. Arrows: megakaryocyte, triangle: adipocyte, scale bar: 100 μm. (**D**) Megakaryocyte counts in images of H&E-stained femurs. Three fields were randomly selected in each sample, *n* = 3. Student’s *t*-test or one-way ANOVA test was performed to compare between two groups or multiple groups, respectively. * *p* < 0.05, ** *p* < 0.01, **** *p* < 0.0001, compared with sham-irradiation. # *p* < 0.05, ## *p* < 0.01, compared with the WT mice. (**E**–**G**) WT and RNase L^−/−^ mice were exposed to 6 Gy of TBI. WBCs (**E**), RBCs (**F**), and PLTs (**G**) were analyzed in peripheral blood, *n* = 5. Two-way ANOVA test was performed for comparison. * *p* < 0.05, ** *p* < 0.01, compared with the WT mice.

To investigate the potential involvement of RNase L in irradiation-induced body injury, we exposed the wildtype (WT) and RNase L^−/−^ mice to 6 Gy of TBI and assessed the corresponding phenotypes (Figure 1A). Firstly, we evaluated the changes in body weight of the mice four weeks after irradiation. The body weight of mice decreased within 4 d after irradiation, followed by subsequent recovery. The RNase L^−/−^ mice exhibited higher body weight compared with the WT mice (Figure 1B). Furthermore, histopathology analysis of the bone marrow revealed a mild injury to hematopoietic cells at 2 and 6 h, a severe depletion of various hematopoietic cells at 24 h, an increase in mononuclear cells and a sustained reduction in megakaryocytes which were replaced by adipocytes at 10 d after irradiation (Figure 1C). Notably, the bone marrow in RNase L^−/−^ mice exhibited less damage to hematopoietic cells at 24 h and improved recovery at 10 d after irradiation compared with the WT mice (Figure 1C). Irradiation significantly decreased the number of bone marrow megakaryocytes, but this decrease was reversed in RNase L^−/−^ mice at both 24 h (*p* < 0.01) and 10 d (*p* < 0.05) as shown in Figure 1D. Consistent with the histopathological findings, the peripheral blood count demonstrated that irradiation caused significant depletion of white blood cells (WBCs), red blood cells (RBCs), and platelets (PLTs). However, RNase L deficiency mitigated the depletion of WBCs at 24 h after irradiation (Figure 1E, *p* < 0.05), ameliorated the damage to RBCs (Figure 1F), and partly alleviated the decrease in PLTs caused by irradiation (Figure 1G). These results suggested that RNase L deficiency partially alleviates irradiation-induced body injury, indicating the involvement of RNase L in the development of irradiation-induced bone marrow damage.

### 2.2. Effect of RNase L Knockout on Gene Expression in Bone Marrow Cells

To investigate the potential mechanism of RNase L in irradiation-induced bone marrow injury, RNA-seq analysis was performed on bone marrow cells collected from WT and RNase L^−/−^ mice exposed to 6 Gy of TBI or sham-irradiation. Pearson correlation analysis revealed a significant correlation with correlation coefficients exceeding 0.96 across most samples, except for the RNase L^−/−^-2 sample (Figure S1A). The RNase L^−/−^-2 sample was excluded from the subsequent bioinformatic analysis due to its apparent dissimilarity from the other samples, while the RNase L^−/−^-1 and RNase L^−/−^-3 samples exhibited good reproducibility. The clustering result showed both intragroup similarities and intergroup differences (Figure S1B).

**Figure 2:**
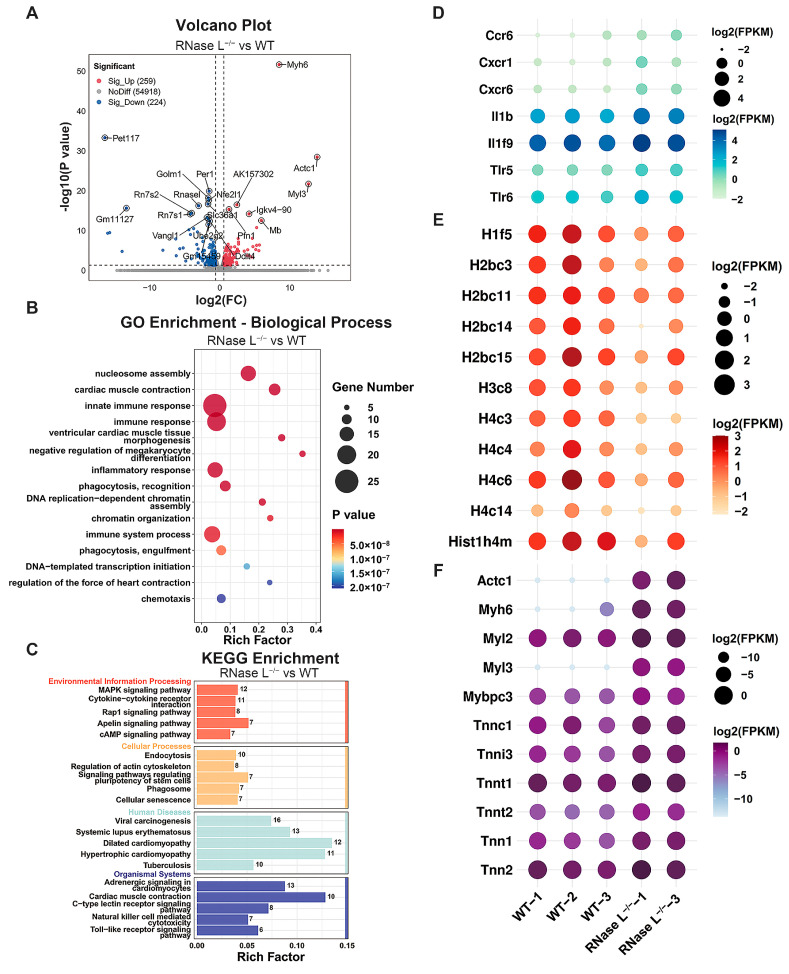
Effect of RNase L knockout on gene expression in the bone marrow cells. (**A**) Volcano map shows the DEGs between the RNase L^−/−^ and WT groups. The upregulated or downregulated DEGs are colored in red or blue, and the undifferentiated genes expressed in both groups are colored in gray. (**B**,**C**) GO biological process (**B**) and KEGG (**C**) analysis for DEGs in (**A**). The numbers in each column indicate the number of DEGs. (**D**–**F**) Bubble plot shows the expression levels of selected genes in GO terms of innate immune response (**D**), nucleosome assembly (**E**), and cardiac muscle contraction (**F**).

We first investigated the impact of RNase L deficiency on biological functions in the bone marrow. Differential gene expression analysis revealed that 259 genes were upregulated, and 224 genes were downregulated between the RNase L^−/−^ and WT groups (Figure 2A). Gene Ontology (GO) biological process analysis indicated that the differentially expressed genes (DEGs) were significantly enriched in nucleosome assembly, cardiac muscle contraction, and innate immune response terms (Figure 2B), then cellular component or molecular function analysis showed these DEGs were localized to the membrane and cytoplasm or involved in protein binding, respectively (Figure S2A and Table S1). Consistent with GO analysis, Kyoto Encyclopedia of Genes and Genome (KEGG) analysis revealed these DEGs were enriched in cytokine–cytokine receptor interaction, regulation of actin cytoskeleton, and dilated cardiomyopathy pathways (Figure 2C and Table S1). Furthermore, the expression of immune response-related genes such as C-C motif chemokine receptor 6 (*Ccr6*), C-X-C motif chemokine receptor 1 (*Cxcr1*), *Il1b*, toll-like receptor 5 (*Tlr5*), was significantly upregulated in the RNase L^−/−^ group compared with the WT group (Figure 2D). These findings indicated that RNase L deficiency disrupts the expression of cytokine-related and toll-like receptor-related genes in the bone marrow. A previous study reported that RNase L deficiency disrupts gastrointestinal homeostasis by triggering chronic inflammation [32]. RNase L deficiency may also affect the immune homeostasis in the bone marrow. Histone-related genes involved in nucleosome assembly term were significantly downregulated in the RNase L^−/−^ group (Figure 2E). Additionally, cardiac muscle contraction-related genes including actin alpha cardiac muscle 1, myosin heavy polypeptide 6 (*Myh6*), troponin C1, cardiac/slow skeletal (*Tnnc1*), and so on, were significantly upregulated in the RNase L^−/−^ group (Figure 2F). It has been reported that increasing RNase L activity downregulates the mRNA expression of myogenic differentiation 1 (*MyoD1*), indicating the involvement of RNase L in myoblast differentiation [33]. Overall, these findings suggested that RNase L affects multiple biological functions by regulating the expression of special genes associated with cytokines, histones, and myosin in the bone marrow.

### 2.3. The Expression of Rnase L and Oas Family Genes Is Upregulated after Irradiation

**Figure 3:**
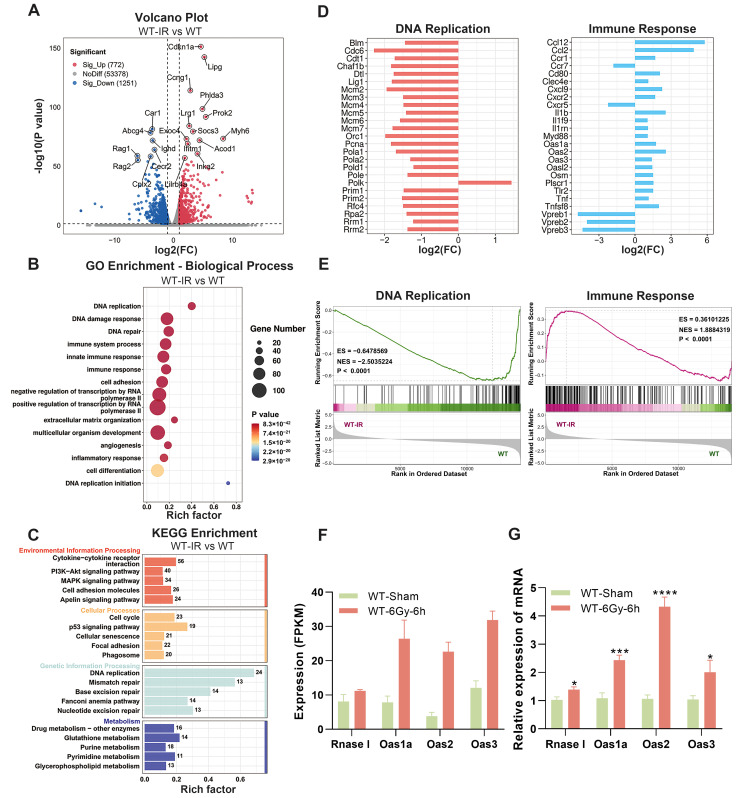
The expression of *Rnase l* and Oas family genes is upregulated after irradiation. (**A**) Volcano map shows the DEGs between the WT-IR and WT groups. The upregulated or downregulated DEGs are colored in red or blue, and the undifferentiated genes expressed in both groups are colored in gray. (**B**,**C**) GO biological process (**B**) and KEGG (**C**) analysis for the DEGs in (**A**). Numbers in the column indicate the number of DEGs. (**D**) The expression levels of the top 25 genes in GO terms of DNA replication and immune response. (**E**) GSEA displays DNA replication and immune response terms differentially enriched in the WT and WT-IR groups. (**F**,**G**) Histogram of the expression levels of *Rnase l*, *Oas1a*, *Oas2*, and *Oas3* measured by RNA-seq (**F**) and qRT-PCR (**G**). For qRT-PCR, actin beta (*Actb*) was used as the reference gene, *n* = 6. Student’s *t*-test was performed to compare between two groups. * *p* < 0.05, *** *p* < 0.001, **** *p* < 0.0001.

To further investigate the role of RNase L in the bone marrow after irradiation, we analyzed the DEGs between the WT-IR and WT groups, as well as between the RNase L^−/−^-IR and RNase L^−/−^ groups. We first analyzed the DEGs between the WT-IR and WT groups and revealed that 772 genes were upregulated and 1251 genes were downregulated (Figure 3A). GO biological process analysis demonstrated significant enrichment of these DEGs in DNA replication, DNA repair, and immune-related terms, including immune system process, innate immune response, and immune response (Figure 3B). Cellular component or molecular function analysis indicated that these DEGs were localized to the membrane and cytoplasm or involved in protein binding and DNA binding, respectively (Figure S2B and Table S2). Consistent with GO analysis, KEGG analysis revealed enrichment in pathways related to the cell cycle, p53 signaling, and DNA replication and repair (Figure 3C and Table S2). Furthermore, genes involved in DNA replication exhibited significant downregulation, while immune response-related genes displayed significant upregulation after irradiation (Figure 3D). Gene Set Enrichment Analysis (GSEA) indicated that DNA replication was significantly enriched in the WT group compared with the WT-IR group, whereas immune response was enriched in the WT-IR group compared with the WT group (Figure 3E). These results demonstrated that irradiation induces a defect in DNA replication and activates the immune responses. Existing research reported that irradiation impacts the expression of genes involved in DNA replication and repair, cell cycle, and p53 pathway in the bone marrow [34]. Notably, genes (*Oas1a*, *Oas2*, *Oas3*, *Oasl2*) belonging to the Oas family in the immune response term were significantly upregulated after irradiation (Figure 3C). RNA-seq and qRT-PCR analysis further demonstrated that the expression of *Rnase l*, *Oas1a*, *Oas2*, and *Oas3* genes was upregulated after irradiation (Figure 3F,G). These results suggested the potential involvement of the OAS/RNase L pathway in the irradiation-induced immune response.

### 2.4. Effect of RNase L Knockout on Gene Expression in Bone Marrow Cells after Irradiation

**Figure 4:**
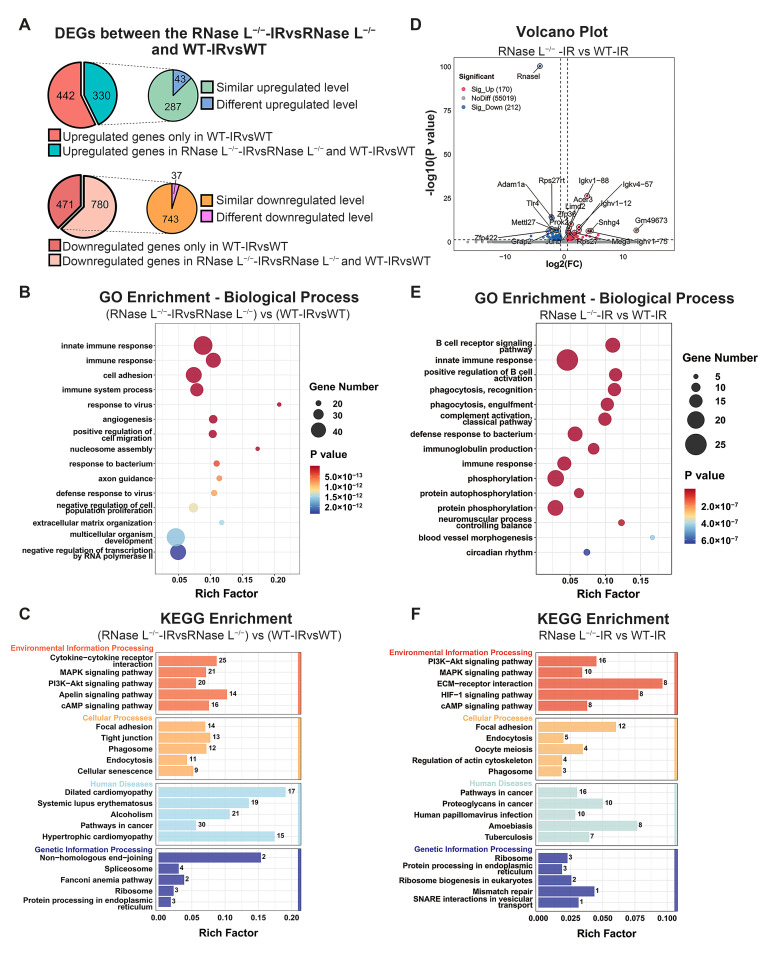
RNase L knockout affects multiple pathways in the bone marrow after irradiation. (**A**) Pie graphs display that 772 or 1251 genes are upregulated or downregulated between the WT-IR and WT groups, respectively. However, 442 or 471 genes are not upregulated or downregulated between the RNase L^−/−^-IR and RNase L^−/−^ groups. Moreover, 43 or 37 genes were different in the expression level of upregulated or downregulated between the RNase L^−/−^-IR and RNase L^−/−^ groups compared with between the WT-IR and WT groups. (**B**,**C**) GO biological process (**B**) and KEGG (**C**) analysis for these 993 DEGs in (**A**). Numbers in the column indicate the number of DEGs. (**D**) Volcano map shows the DEGs between the RNase L^−/−^-IR and WT-IR groups. The upregulated or downregulated DEGs are colored in red or blue, and the undifferentiated genes expressed in both groups are colored in gray. (**E**,**F**) GO biological process (**E**) and KEGG (**F**) analysis for the DEGs in (**D**). Numbers in the column indicate the number of DEGs.

To explore the genes affected by RNase L knockout after irradiation, we compared 772 upregulated genes and 1251 downregulated genes between the WT-IR and WT groups (Figure 3A), with the DEGs between the RNase L^−/−^-IR and RNase L^−/−^ groups. Among these, 330 or 780 genes were also upregulated or downregulated in the RNase L^−/−^-IR group compared with the RNase L^−/−^ group, whereas 442 or 471 genes showed no significant upregulation or downregulation (Figure 4A and Table S3). Moreover, 43 or 37 genes were different in the expression level of upregulated or downregulated between the RNase L^−/−^-IR and RNase L^−/−^ groups compared with between the WT-IR and WT groups among the 330 or 780 genes upregulated or downregulated in both comparisons (Figure 4A and Table S3). These findings suggested that RNase L knockout affects the expression of these 993 genes after irradiation. GO biological process analysis indicated these 993 genes were significantly enriched in terms related to immune responses and cell adhesion (Figure 4B), then cellular component or molecular function analysis revealed these genes were localized to membrane and cytoplasm or involved in protein binding, respectively (Figure S2C and Table S4). Consistent with GO analysis, KEGG analysis demonstrated these genes were enriched in cytokine–cytokine receptor interaction, PI3k-Akt signaling, and focal adhesion pathways (Figure 4C and Table S4). The above analysis is based on the DEGs induced by irradiation. To further supplement the DEGs between the WT and RNase L^−/−^ mice exposed to irradiation, we analyzed the DEGs between the RNase L^−/−^-IR and WT-IR groups and found 170 genes were upregulated and 212 genes were downregulated (Figure 4D). GO biological process analysis demonstrated that the DEGs were significantly enriched in B cell regulation-related terms (Figure 4E), and cellular component or molecular function analysis revealed these DEGs were localized to membrane and cytoplasm or involved in protein binding, respectively (Figure S2D and Table S5). Consistent with GO analysis, KEGG analysis showed these DEGs were enriched in PI3K-Akt signaling pathway and ECM-receptor interaction (Figure 4F and Table S5). Collectively, the DEGs exhibited significant enrichment in terms related to immune response and B cell regulation, as well as in the PI3K-Akt signaling and ECM-receptor interaction pathways.

**Figure 5:**
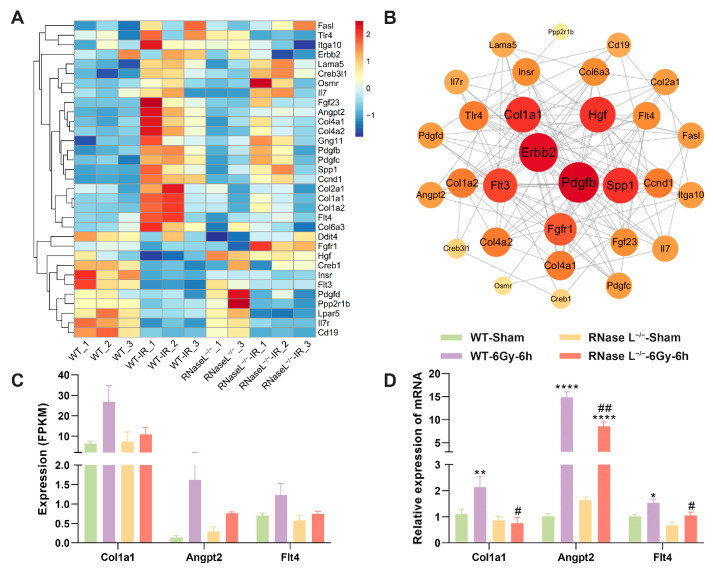
RNase L deficiency counteracts the upregulation of genes associated with the PI3K-Akt pathway induced by irradiation. (**A**) Heat map shows the expression of DEGs in the PI3K-Akt pathway from KEGG analysis. (**B**) PPI analysis displays the interaction between the DEGs in the PI3K-Akt pathway. (**C**,**D**) Histogram of the expression levels of selected genes in PI3K-Akt pathway measured by RNA-seq (**C**) and qRT-PCR (**D**). For qRT-PCR, *Actb* was used as the reference gene, *n* = 6. Student’s *t*-test was performed to compare between two groups. * *p* < 0.05, ** *p* < 0.01, **** *p* < 0.0001, compared with sham-irradiation. # *p* < 0.05, ## *p* < 0.01, compared with the WT mice.

Furthermore, we analyzed the DEGs in the PI3K-Akt signaling pathway from the above analyses. The heat map showed the expression of most of the DEGs was notably downregulated in the RNase L^−/−^-IR group compared with the WT-IR group (Figure 5A). Protein-Protein Interaction (PPI) analysis revealed a strong interconnection between the DEGs, with collagen type I alpha 1 (*Col1a1*) and erb-b2 receptor tyrosine kinase 2 exhibiting higher degrees of connectivity (Figure 5B). Additionally, the expression of selected genes including *Col1a1*, angiopoietin 2 (*Angpt2*), and FMS-like tyrosine kinase 4 (*Flt4*) was significantly upregulated after irradiation, whereas RNase L deficiency inhibited the increase verified by RNA-seq (Figure 5C) and qRT-PCR analyses (Figure 5D). These findings suggested that RNase L deficiency counteracts the upregulation of genes induced by irradiation associated with the PI3K-Akt signaling pathway.

### 2.5. RNase L Deficiency Inhibits the Upregulation of Immune Response-Related Genes after Irradiation

**Figure 6:**
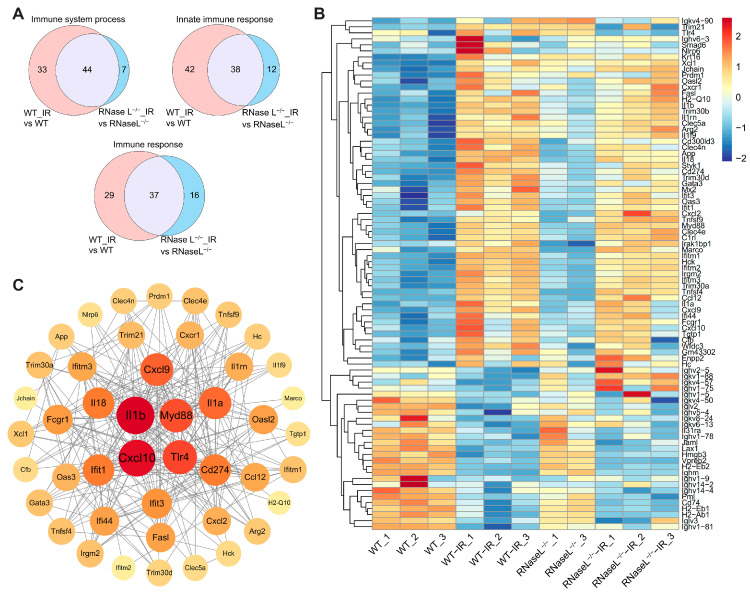
The DEGs in the immune response-related terms. (**A**) Venn diagrams show the common or different genes in the immune response-related terms between the RNase L^−/−^-IR and RNase L^−/−^ groups compared with between the WT-IR and WT groups. (**B**) Heat map shows the expression of the DEGs in immune response-related terms from GO analysis. (**C**) PPI analysis displays the interaction between the upregulated DEGs in immune response-related terms.

**Figure 7:**
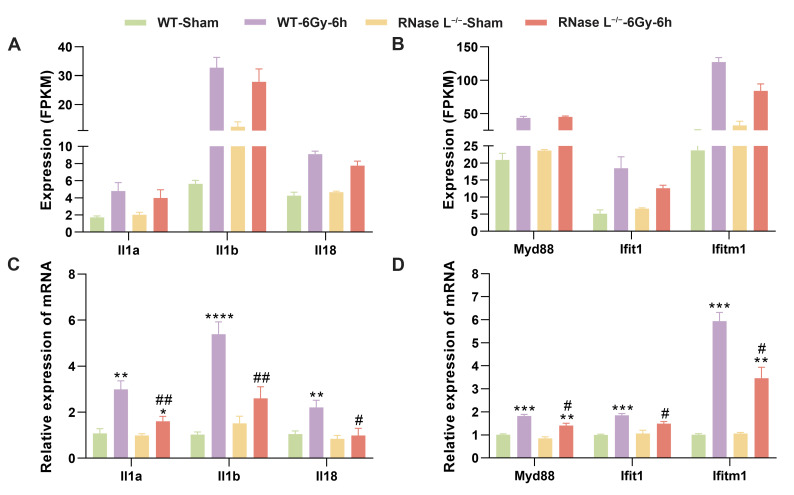
RNase L deficiency inhibits the upregulation of genes related to the immune response after irradiation. (**A**–**D**) Histogram of the expression levels of proinflammatory cytokine and interferon-stimulated genes measured by RNA-seq (**A**,**B**) and qRT-PCR (**C**,**D**). For qRT-PCR, *Actb* was used as the reference gene, *n* = 6. Student’s *t*-test was performed to compare between two groups. * *p* < 0.05, ** *p* < 0.01, *** *p* < 0.001, **** *p* < 0.0001, compared with sham-irradiation. # *p* < 0.05, ## *p* < 0.01, compared with the WT mice.

Based on the DEGs between the RNase L^−/−^-IR and RNase L^−/−^ groups compared with between the WT-IR and WT groups, they were significantly enriched in immune response-related terms, including immune system process, innate immune response, and immune response (Figure 4). The Venn diagrams illustrated a decrease in the number of DEGs related to the immune responses between the RNase L^−/−^-IR and RNase L^−/−^ groups, suggesting that RNase L deficiency attenuates changes in immune-related genes (Figure 6A). Further analysis revealed 58 upregulated and 27 downregulated DEGs between the two groups within the three immune response-related terms (Figure 4B and Table S4). The heat map displayed the expression patterns of these 85 DEGs, indicating that the majority of the upregulated DEGs induced by irradiation exhibited a lower degree of upregulation in the RNase L^−/−^-IR group compared with the WT-IR group (Figure 6B). Subsequent PPI analysis of these 58 upregulated DEGs demonstrated a strong interconnection, with *Il1b* and myeloid differentiation primary response gene 88 (*Myd88*) exhibiting higher degrees of connectivity (Figure 6C). Notably, previous studies have reported an interregulation between RNase L and type I interferon [35], and the activation of RNase L leading to increased expression of cytokines such as IL1B during pathogenic microorganism invasion [36]. Therefore, we selected several genes of the cytokines and interferon-stimulated genes for further validation. RNA-seq analysis demonstrated the expression of proinflammatory cytokine genes (e.g., *Il1a*, *Il1b*, *Il18*), interferon-stimulated genes (e.g., interferon-induced protein with tetratricopeptide repeats 1 [*Ifit1*], interferon-induced transmembrane protein 1 [*Ifitm1*]), and *Myd88* was upregulated after irradiation, whereas RNase L deficiency significantly inhibited their upregulation (Figure 7A,B). The verification of gene expression through qRT-PCR coincided with the findings from the RNA-seq analysis (Figure 7C,D). These findings suggested that RNase L deficiency inhibits the upregulation of immune response-related genes, including proinflammatory cytokine and interferon-stimulated genes. The reduction of proinflammatory cytokines and interferon-stimulated genes alleviates the inflammatory response, thereby mitigating irradiation-induced bone marrow injury in RNase L^−/−^ mice.

### 2.6. RNase L Regulates Irradiation-Induced Immunoglobulins Expression

**Figure 8:**
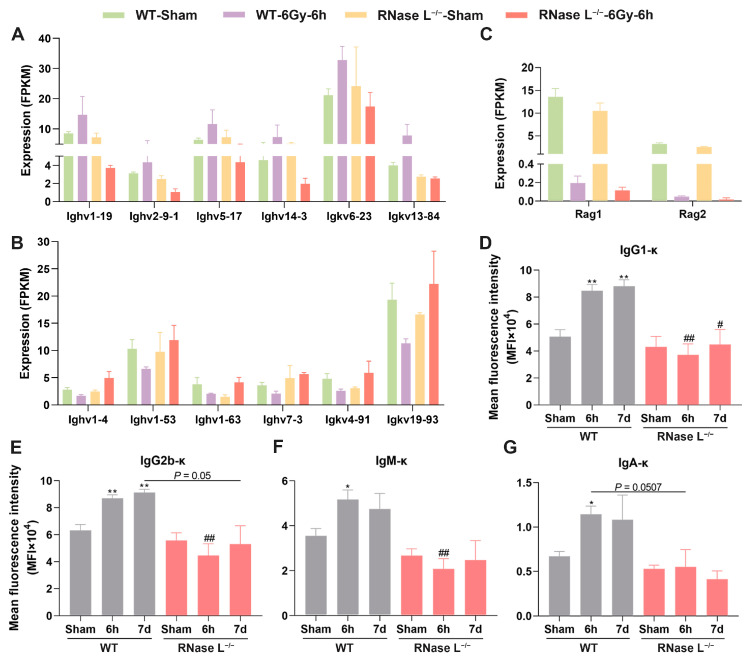
RNase L regulates irradiation-induced immunoglobulins expression. (**A**,**B**) Histogram of the gene expression of the B cell regulation-related terms measured by RNA-seq. (**C**) Histogram of the gene expression of V(D)J-recombinase measured by RNA-seq. (**D**–**G**) CBA analysis shows the levels of κ chain of IgG1 (**D**), IgG2b (**E**), IgA (**F**), and IgM (**G**) in serum of the WT and RNase L^−/−^ mice treated with 6Gy of TBI or sham-irradiation (*n* = 3). Student’s *t*-test or one-way ANOVA test was performed to compare between two groups or multiple groups, respectively. * *p* < 0.05, ** *p* < 0.01, compared with sham-irradiation. # *p* < 0.05, ## *p* < 0.01, compared with the WT mice.

Based on the DEGs between the RNase L^−/−^-IR and WT-IR groups that were significantly enriched in B cell regulation-related terms (Figure 4E), further analysis of the DEGs revealed that they primarily consisted of genes associated with immunoglobulin heavy chain variable region (*Ighv*) and immunoglobulin kappa chain complex variable region (*Igkv*) in B cell regulation-related terms. Notably, RNA-seq analysis demonstrated the majority of *Ighv* and *Igkv* genes were dysregulated in the bone marrow of WT mice after irradiation. Specifically, two distinct patterns were observed, with some genes of *Ighv* and *Igkv* being upregulated (Figure 8A) and others being downregulated (Figure 8B) in response to irradiation, while RNase L deficiency partly reversed this dysregulation. A previous study reported that exposure to gamma radiation increases the frequency of V(D)J recombination in a dose-dependent manner [37]. Specifically, the expression of V(D)J recombinase recombination activating 1 (*Rag1*) and recombination activating 2 (*Rag2*), which mediate rearrangements, was found to significantly decrease in the bone marrow after irradiation; however, no significant difference was observed between the RNase L^−/−^-IR and WT-IR groups (Figure 8C). These findings suggested that irradiation disrupts the expression of genes associated with *Rag1*, *Rag2*, *Ighv*, and *Igkv*, indicating a disturbance of the V(D)J rearrangement. Additionally, it was observed that RNase L deficiency may alter the gene expression pattern of *Ighv* and *Igkv*. According to the report, the expression of Rag1 and Rag2 was downregulated leading to the suppression of V(D)J recombination in immature lymphocytes following DNA damage caused by irradiation, which is dependent on ATM kinase [38]. Furthermore, we detected the levels of immunoglobulin isotyping in the serum of WT and RNase L^−/−^ mice after irradiation. Interestingly, the levels of κ chain of IgG1 (Figure 8D), IgG2b (Figure 8E), IgA (Figure 8F), and IgM (Figure 8G) were increased at 6 h and 7 d after irradiation, while RNase L deficiency significantly inhibited the increase induced by irradiation. These results indicated that RNase L plays a role in regulating the expression of immunoglobulins induced by irradiation. RNase L deficiency may partially alleviate hyperactivated immune responses by inhibiting the upregulation of immunoglobulins in the serum, thereby reducing irradiation-induced bone marrow damage.

## 3. Discussion

**Figure 9:**
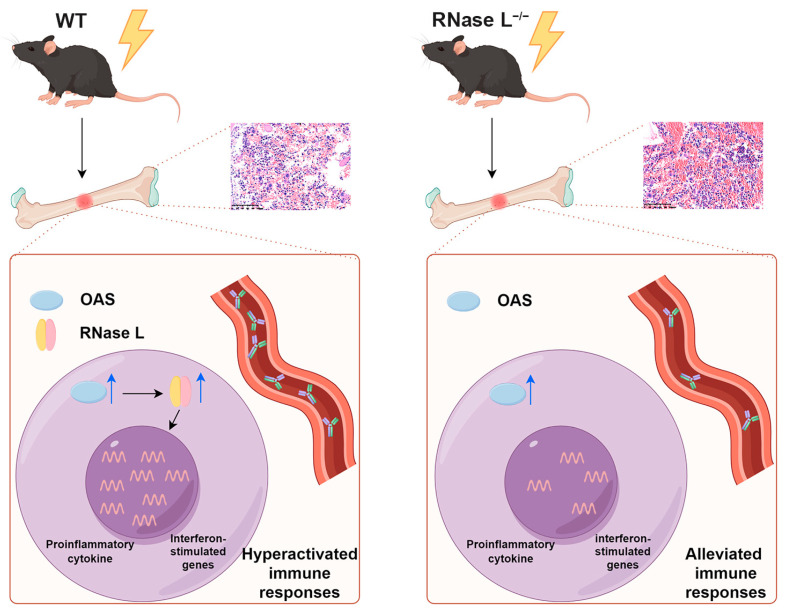
RNase L deficiency alleviates irradiation-induced immune response activation in the bone marrow. The blue arrows indicate upregulated expression levels. The black arrows indicate promotion. This figure was generated by Figdraw 2.0.

The activation of the RNase L pathway and subsequent induction of the immune responses occur in response to viral infection or cellular stress in the absence of pathogens. RNase L is crucial for maintaining intracellular homeostasis. This study presents the novel role of RNase L in the immune response induced by irradiation in the bone marrow. First, we observed that the absence of RNase L partially mitigates irradiation-induced bone marrow injury in mice. Then, RNase L deficiency alleviates the irradiation-induced immune response by modulating the expression of cytokines and interferon-stimulated genes. RNase L deficiency counteracts the increased levels of κ chain of IgG1, IgG2b, IgA, and IgM induced by irradiation in serum, thereby partially alleviating hyperactivated immune responses. Thus, RNase L deficiency alleviates irradiation-induced bone marrow injury by mitigating the immune responses (Figure 9). These findings suggest that RNase L plays a role in the immune response induced by irradiation in the bone marrow.

A comparison between the WT and RNase L^−/−^ mice revealed a significant downregulation of histone-related genes in the absence of RNase L. Previous research reported that ABCE1 (also named RNase L inhibitor) is crucial for the progression of the S phase through its regulation of histone biosynthesis and DNA replication; however, the involvement of RNase L in this process remains unknown [39]. Notably, irradiation leads to downregulation of histone expression [40], which in turn promotes DNA damage repair [41,42]. Our previous study demonstrated that RNase L interacts with x-ray repair cross complementing 4 and DNA ligase 4, both of which are involved in DNA end-joining, and facilitates DNA DSB repair [43]. Consequently, it is suggested that RNase L may play a role in the regulation of genome stability. Further investigation is necessary to examine the regulatory relationship between RNase L and histones. Moreover, RNase L deficiency leads to a significant upregulation of genes (e.g., *Myh6*, *Tnnc1*) associated with myosin and troponin. A previous study reported that RNase L plays a pivotal role in determining muscle cell fate via regulating gene expression during myogenesis, overexpression of RNase L downregulates the expression of MyoD1 which promotes myogenesis, and the expression of Aebp1 and Chop-10/Ddit3 which inhibit adipogenesis, then inhibits myogenesis and favors adipogenesis [44]. Notably, myosin heavy chain 9, troponin I, and clathrin light chain beta can interact with RNase L in mouse spleen [45]. These findings suggest that RNase L may be involved in biological functions by potentially influencing the expression of myosin and troponin either directly or indirectly.

In our results, the expression of *Rnase l*, *Oas1a*, *Oas2*, and *Oas3* genes was upregulated after irradiation. As reported, the expression of RNase L is upregulated after microwave exposure [46]. The upregulation of OAS1, OAS3, and IFITM1 expression in various tumor cell lines is observed following multi-fraction irradiation [47]. These results indicate the potential involvement of the OAS-RNase L pathway in irradiation. Phenotypic analysis of WT and RNase L^−/−^ mice revealed that RNase L knockout partially relieves irradiation-induced body injury. Additionally, RNA-seq analysis of the bone marrow demonstrated that RNase L deficiency inhibits the increased expression of proinflammatory cytokines (*Il-1a*, *Il-1b*, *Il-18*), interferon-stimulated genes (*Ifit1*, *Ifitm1*), and *Myd88* induced by irradiation. Previous studies have reported that the elimination of RNase L suppresses the increased expression of IL1B, IL4, and IL10, and the activation of the TLR4 pathway in macrophages stimulated by LPS [48], and reduces IL1B production in mice infected by the influenza A virus [36]. Furthermore, the synergistic effect of IFIT, IFNB, and the OAS/RNase L pathway on virus resistance has been documented [49]. Myd88, functioning as a signal transducer, can induce the expression of multiple proinflammatory factors; it has been reported that antibiotic pretreatment alleviates intestinal inflammation induced by irradiation via inhibiting the TLR4-MyD88-NF-κB signaling pathway [50]. Consequently, RNase L deficiency modulates cytokines expression, thereby diminishing the inflammatory response and the bone marrow injury triggered by irradiation. Moreover, several immune-related genes exhibited similar regulatory patterns in both the WT and RNase L^−/−^ mice, indicating that RNase L does not significantly impact the expression of these genes post-irradiation. This finding implied that the RNase L pathway, along with other pathways such as TLR [51] and cGAS-STING [10] signaling pathways, collectively contributes to the modulation of the immune responses triggered by irradiation. The distinct roles of these pathways in regulating a common physiological function are essential for the organism’s ability to effectively respond to external stimuli.

Furthermore, the gene expression of *Ighv* and *Igkv* was dysregulated in the bone marrow after irradiation. As reported, irradiation exposure increases the mutation frequency of *Ighv* in patients with chronic lymphocytic leukemia [52]. Clean-up workers in the Chornobyl accident exhibited an increased usage of IgHV1–69 (orthologs with *Ighv1–4*, *Ighv1–53*, *Ighv1–63* in mice) and IgHV3–21 (orthologs with *Ighv5–17* in mice) genes [53]. Our results revealed that *Ighv1–4*, *Ighv1–53*, *Ighv1–63*, and *Ighv5–17* were dysregulated after radiation in the bone marrow. Notably, the levels of κ chain of IgG1, IgG2b, IgA, and IgM were elevated at 6 h and 7 d after radiation, but this increase was significantly reversed by RNase L deficiency. A previous study has reported that the upregulation of κ chain expression, which is dependent on G2 arrest, is triggered by the accumulation of p53 protein following irradiation [54]. In beagle dogs, the IgA level exhibited a significant increase at 10 d after the lowest dose (4.25–4.65 Gy) of TBI, while the IgG1 level was significantly decreased [55]. The heightened expression of IgG, IgM, and IgA is associated with the hyperactivated immune responses induced by irradiation, resulting in an excessive inflammatory response and body injury. Thus, RNase L deficiency reduces the expression of immunoglobulins induced by irradiation, thereby mitigating the immune responses. As reported, the activation of RNase L results in a significant increase in the fraction of translating ribosomes within open reading frames (ORFs) of coding sequences and ORFs within 5′ and 3′ untranslated regions, suggesting that mRNA decay fragments are translated to produce short peptides that may be recognized by the adaptive immune system, promote immune responses and antiviral activity [56]. Notably, the balance between physiological levels and lower levels of RNase L is critical for maintaining cell homeostasis and ensuring an appropriate response to infection. While the protective function of RNase L necessitates its presence at physiological levels, excessive activity of this enzyme can be detrimental to cells by causing degradation of cellular RNA and inhibition of protein synthesis [25].

Notably, our observations indicated that the gene expression of the PI3K pathway (e.g., *Col1a1*, *Angpt2*, *Flt4*, etc.) is upregulated induced by irradiation, whereas RNase L deficiency reverses the increase. Existing research reported that the hyperactive PI3K delta isoform, which is the primary and most abundant in immune cells, is related to damaged immunization responses and abnormal levels of serum immunoglobulins, including elevated IgM and varying levels of IgG and IgA [57]. This study suggests that the PI3K pathway is essential for regulating the function of immune cells. Furthermore, a previous study reported that RNase L deficiency attenuates macrophage functions, which could be caused by inhibiting the PI3K pathway, and suggests RNase L plays a role in innate immunity by regulating macrophage functions [58]. Therefore, it is necessary to further investigate whether RNase L also regulates B cell function following irradiation. Then, we also found the expression of *Cxcr1* and *TLR5* significantly upregulated in the RNase L^−/−^ mice compared with the WT mice. A previous study reported that the level of antigen-specific IgG1 is elevated in the serum of Cxcr1^−/−^ mice during the thymus-dependent antibody responses [59]. TLR5^+^ Lamina propria dendritic cells induced the differentiation of naive B cells into plasma cells which produced IgA through TLR5 stimulation against bacterial infection in the gut [60]. Thus, chemokines and pattern-recognition receptors are also important for regulating adaptive immunity. The immune responses is a highly intricate regulatory network, including the regulation of various cytokines, diverse immune cell types, and multiple pathways. It is worth noting that RNase L has been involved in the immune response triggered by irradiation in the bone marrow. However, the exact regulatory mechanism remains unclear. During viral infections, RNase L has been demonstrated to promote NLRP3 inflammasome activation through a signaling pathway involving DHX33 and MAVS, leading to the production of IL-1B in bone marrow-derived dendritic cells [36]. Moreover, RNase L has been reported to regulate the function of bone marrow-derived macrophages (BMMs) in vitro, with RNase L deficiency resulting in impaired migration of BMMs in response to M-CSF stimulation and altered expression of key inflammatory genes such as IL-1B, IL-10, M-CSF, and CCL2 [58]. Therefore, the identification of the exact cells and pathways by which RNase L modulates immune response triggered by irradiation is of considerable scientific importance.

In summary, RNase L is involved in irradiation-induced immune response in the bone marrow by regulating the expression of cytokines and immunoglobulins. RNase L appears to be a molecular switch that regulates the immune response. Irradiation induces RNA damage [13] and triggers the expression of type I interferon. Consequently, the OAS-RNase L pathway is activated after recognizing the abnormal RNA and responding to interferon signals. The activation of RNase L causes substantial degradation of RNA, thereby facilitating the expression of proinflammatory cytokines and contributing to the immune response induced by irradiation. However, when RNase L is knocked out, its activation is impeded, resulting in a partial inhibition of proinflammatory cytokines expression and a mitigation of the immune response caused by irradiation. Activation of RNase L not only facilitates the inflammatory response and induces cell death to preserve homeostasis during pathogen invasion, but also has the potential to trigger autoimmune hyperactivation and inflict harm upon normal cells and tissues under conditions of stress. Moreover, this investigation expands our understanding of the biological roles of RNase L in the context of irradiation-induced bone marrow damage, thereby offering a novel therapeutic approach for managing such injuries by modulating the activation of RNase L.

## 4. Materials and Methods

### 4.1. Mice

WT and RNase L^−/−^ mice on the C57BL/6N genetic background were generated by Cyagen Bioscience (Suzhou, China). Male mice aged 7–9 weeks were used in this study. The mice were maintained in a specific pathogen-free environment under a 12/12 h light–dark cycle. All experimental procedures conducted in this study were approved by the Institutional Animal Care and Use Committee at the Animal Center in the Academy of Military Medical Science (Beijing, China) under permit number IACUC-DWZX-2020-541.

### 4.2. Irradiation Exposure and Bone Marrow Extraction

The mice were divided into four groups: (1) WT: WT mice without irradiation, (2) WT-IR: WT mice that underwent irradiation, (3) RNase L^−/−^: RNase L^−/−^ mice without irradiation, and (4) RNase L^−/−^-IR: RNase L^−/−^ mice that underwent irradiation. The mice were placed in individual chambers of well-ventilated plexiglass irradiation boxes, then performed TBI with a single dose of 6 Gy using a ^60^Co gamma-ray source at the Beijing Institute of Radiation Medicine (Beijing, China), while the sham groups were placed in the boxes with no irradiation treatment (Figure 1A). The dose rate of all irradiation exposure was 61.77–77.66 cGy/min in this study. Subsequently, the femurs and tibiae of mice were dissected and cleaned of surrounding tissue at different times after irradiation. The distal and proximal ends were opened, and the bone marrow was flushed using ice-cold phosphate-buffered saline. Finally, the bone marrow cells were collected following centrifugation.

### 4.3. Bone Marrow Histology and Complete Blood Count

The femurs were obtained from three mice per group at 2 h, 6 h, 24 h, and 10 d after irradiation or sham-irradiation, and then fixed, decalcified, and paraffin-embedded. Furthermore, the sections (3 μm) were prepared and stained with hematoxylin and eosin. Images of the bone marrow sections were captured by Digital Pathology Slide Scanner (KFBIO, Ningbo, China). Peripheral blood was collected from the tail veins of the mice and immediately mixed with the diluent at 2 d before irradiation and 1, 4, 7, 10, 14, 18, 22, and 28 d after irradiation. The number of blood cells, including WBCs, RBCs, and PLTs, were measured by the automatic blood cell analyzer (DYMIND, Shenzhen, China).

### 4.4. RNA Extraction and RNA Sequencing (RNA-Seq)

Total RNA was extracted from bone marrow cells that were isolated from three mice per group at 6 h after 6 Gy of TBI using Trizol reagent (Invitrogen, Carlsbad, CA, USA), according to the manufacturer’s instructions. The RNA structure integrity and purity were detected using the Bioanalyzer 2100 (Agilent, Santa Clara, CA, USA) with RIN number > 7.0. Poly (A) RNA is purified from 1 μg total RNA using Dynabeads Oligo (dT) (Thermo Fisher Scientific, Waltham, MA, USA) and then reverse-transcribed to create the cDNA by SuperScript™ II Reverse Transcriptase (Invitrogen, Carlsbad, CA, USA). The cDNA libraries were sequenced by LC-Bio Technology Co. (Hangzhou, China) using the Illumina Novaseq^TM^ 6000 sequence platform. The RNA-seq data and gene expression profiles have been uploaded to the public database of Gene Expression Omnibus at https://www.ncbi.nlm.nih.gov/geo/ (assessed on 27 October 2023) under accession number: GSE211710.

### 4.5. Bioinformatic Analysis

DEGs analysis was conducted using the R package edegR between two different groups. A |Fold Change (FC)| > 2 and adjusted *p* value < 0.05 were regarded as significant between the WT-IR and WT groups and between the RNase L^−/−^-IR and RNase L^−/−^ groups, and a |FC| > 1.5 and adjusted *p* value < 0.05 between the RNase L^−/−^ and WT groups and between the RNase L^−/−^-IR and WT-IR groups. GO, KEGG, and GSEA enrichment analyses of DEGs were performed by the OmicStudio tools [61] at https://www.omicstudio.cn/tool (assessed on 22 December 2023). Pearson correlation analysis and hierarchical clustering analysis were also conducted using the OmicStudio tools. The volcano plots, Venn diagrams, bubble diagrams, pie graphs, and heat maps were generated using R version 4.1.3 on the OmicStudio platform. The PPI network analysis used the STRING database [62] at https://cn.string-db.org/ (assessed on 1 November 2023) and Cytoscape v.3.10.0 [63].

### 4.6. Quantitative Real-Time Polymerase Chain Reaction (qRT-PCR) Validation Analysis

Total RNA was extracted from the bone marrow cells isolated from six mice per group at 6 h after 6 Gy of TBI as described above. HiScript III All-in-one RT SuperMix (Vazyme, Nanjing, China) was used to synthesize cDNA. qRT-PCR was performed using TOROGreen^®^ HRM qPCR Master Mix (Torovid, Shanghai, China) and analyzed on Mx3000P QPCR Systems (Agilent, Santa Clara, CA, USA), according to the manufacturer’s instructions. The relative expression of each gene was normalized to actin beta and calculated according to the 2^−∆∆Ct^ method. The primers used for amplifying the target gene are provided in Supplementary Table S6.

### 4.7. Serum Immunoglobulins Level Analysis by Cytometric Bead Array (CBA)

The levels of IgG1, IgG2b, IgA, and IgM in serum were measured using the CBA Mouse Immunoglobulin Isotyping Kit (BD Biosciences, San Jose, CA, USA). Serum samples were collected from three mice per group at 6 h and 7 d after 6 Gy of TBI. The serum diluted 1:100 or the standards diluted 1:10 in the master buffer were respectively mixed with the mouse Ig capture bead array, followed by incubation and centrifugation. The beads were resuspended and mixed with PE/FITC detector antibody mixture, then incubated, centrifuged, and washed. The relative levels of immunoglobulins were determined by calculating the mean fluorescence intensity detected by the flow cytometer (BD Biosciences, San Jose, CA, USA).

### 4.8. Statistical Analysis

GraphPad Prism 8.0 (GraphPad Software, La Jolla, CA, USA) was used for all statistical analyses, and *p* < 0.05 was considered statistically significant. Data are presented as mean ± standard error of the mean. Two-sided student’s *t*-test or one-way ANOVA followed by Dunnett’s multiple comparisons test were used for comparisons between two or multiple groups, respectively. Complete blood count was performed using two-way ANOVA followed by Sidak’s multiple comparisons test for comparisons between the WT and RNase L^−/−^ mice.

## Data Availability

The RNA-seq data and gene expression profiles have been uploaded to the public database of Gene Expression Omnibus at https://www.ncbi.nlm.nih.gov/geo/ (assessed on 27 October 2023) under accession number: GSE211710.

## Supplementary Materials

The following supporting information can be downloaded at: https://www.mdpi.com/article/10.3390/ijms25052722/s1.
