# Supplementary material for: Unraveling the Role of RNase L Knockout in Alleviating Immune Response Activation in Mice Bone Marrow after Irradiation
Source: Int J Mol Sci. 2024 Feb 27;25(5):2722. doi: 10.3390/ijms25052722 (PMC10932110; doi:10.3390/ijms25052722)
Supplement: Supplementary file 1 [file ijms-25-02722-s001.zip › Supplementary figures.pdf]

Supplementary Figures

Unraveling the Role of RNase L Knockout in Alleviating Immune Response Activation in Mice Bone Marrow after Irradiation

Kexin Ding, Hujie Li , Fumin Tai, Junzhao Duan, Qiong Wang, Rui Zhai, Hanjiang Fu, Changhui Ge* and Xiaofei Zheng*

Beijing Key Laboratory for Radiobiology, Department of Experimental Hematology and Biochemistry, Beijing Institute of Radiation Medicine, Beijing 100850, China

***Correspondence**

Xiaofei Zheng, Beijing Key Laboratory for Radiobiology, Department of Experimental Hematology and Biochemistry, Beijing Institute of Radiation Medicine, 27 Taiping Road, Beijing 100850, China

E-mail: zhengxf@bmi.ac.cn

Changhui Ge, Beijing Key Laboratory for Radiobiology, Department of Experimental Hematology and Biochemistry, Beijing Institute of Radiation Medicine, 27 Taiping Road, Beijing 100850, China

E-mail: chge502@163.com

Keywords: RNase L; irradiation; bone marrow; immune response

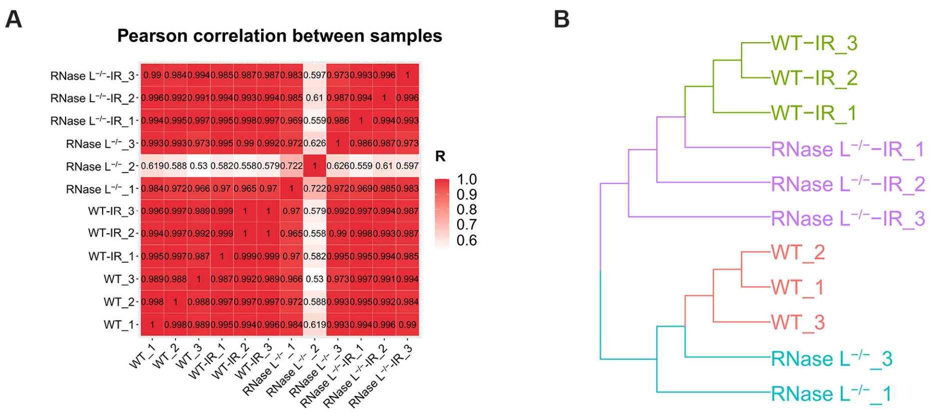

Figure S1. Quality control analysis of RNA-seq data. (A) Pearson analysis shows the correlation between the samples. (B) Hierarchical cluster analysis displays intragroup similarities and intergroup differences.

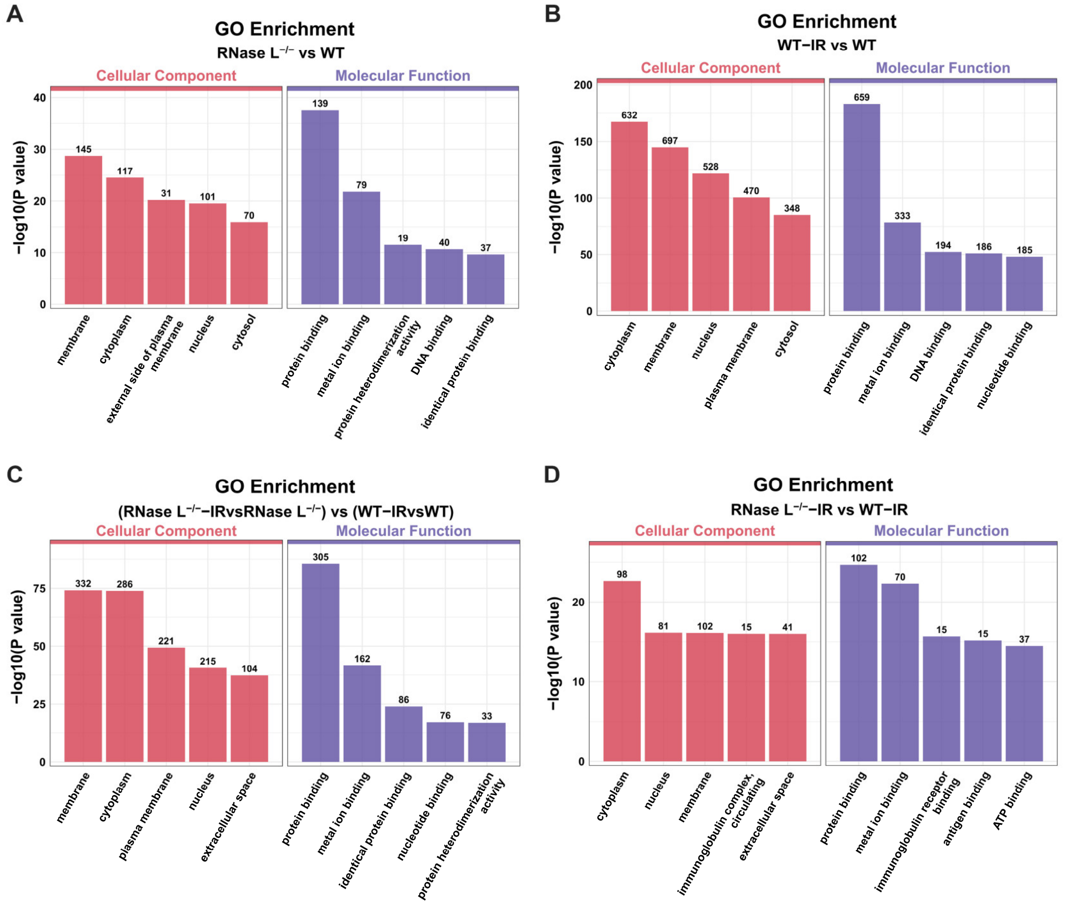

Figure S2. GO analysis based on cellular component and molecular function in different groups. (A) GO analysis between the RNase L$^{-/-}$ and WT groups. (B) GO analysis between the WT-IR and WT groups. (C) GO analysis between the RNase L$^{-/-}$-IR and RNase L$^{-/-}$ groups compared with the WT-IR and WT groups. (D) GO analysis between the RNase L$^{-/-}$-IR and WT-IR groups.
